# Exploring the Temporal Dynamics of the Fungal Microbiome in Rootstocks, the Lesser-Known Half of the Grapevine Crop

**DOI:** 10.3390/jof8050421

**Published:** 2022-04-20

**Authors:** David Gramaje, Aleš Eichmeier, Milan Spetik, María Julia Carbone, Rebeca Bujanda, Jessica Vallance, Patrice Rey

**Affiliations:** 1 Instituto de Ciencias de la Vid y del Vino (ICVV), Consejo Superior de Investigaciones Científicas, Universidad de la Rioja, Gobierno de La Rioja, Ctra. LO-20 Salida 13, Finca La Grajera, 26071 Logroño, Spain; rebeca.bujanda@icvv.es; 2 Faculty of Horticulture, Mendeleum—Institute of Genetics, Mendel University in Brno, Valticka 334, 69144 Lednice, Czech Republic; ales.eichmeier@mendelu.cz (A.E.); milan.spetik@mendelu.cz (M.S.); 3 Departamento de Protección Vegetal, Facultad de Agronomía, Universidad de la República, Montevideo 12900, Uruguay; mariajulia.93@hotmail.com; 4 Bordeaux Sciences Agro, INRAE, ISVV, SAVE, 33140 Villenave d’Ornon, France; jessica.vallance@inrae.fr (J.V.); patrice.rey@agro-bordeaux.fr (P.R.); 5 Université de Bordeaux, Bordeaux Sciences Agro, UMR 1065 SAVE, 33175 Gradignan, France; 6 Institut des Sciences Analytiques et de Physicochimie pour l‘Environnement et les Matériaux—UMR 5254, Université de Pau et des Pays de l’Adour, E2S UPPA, CNRS, IBEAS Avenue de l’Université, 64013 Pau, France

**Keywords:** culture-independent analysis, fungal microbiome, grapevine nursery, grapevine trunk diseases, high-throughput amplicon sequencing, *Vitis vinifera*

## Abstract

Rootstocks are the link between the soil and scion in grapevines, can provide tolerance to abiotic and biotic stresses, and regulate yield and grape quality. The vascular system of grapevine rootstocks in nurseries is still an underexplored niche for research, despite its potential for hosting beneficial and pathogenic microorganisms. The purpose of this study was to investigate the changes in the composition of fungal communities in 110 Richter and 41 Berlandieri rootstocks at four stages of the grapevine propagation process. Taxonomic analysis revealed that the fungal community predominantly consisted of phylum Ascomycota in all stages of the propagation process. The alpha-diversity of fungal communities differed among sampling times for both rootstocks, with richness and fungal diversity in the vascular system decreasing through the propagation process. The core microbiome was composed of the genera *Cadophora*, *Cladosporium*, *Penicillium* and *Alternaria* in both rootstocks, while the pathogenic genus *Neofusicoccum* was identified as a persistent taxon throughout the propagation process. FUNguild analysis showed that the relative abundance of plant pathogens associated with trunk diseases increased towards the last stage in nurseries. Fungal communities in the vascular system of grapevine rootstocks differed between the different stages of the propagation process in nurseries. Numerous genera associated with potential biocontrol activity and grapevine trunk diseases were identified. Understanding the large diversity of fungi in the rootstock vascular tissue and the interactions between fungal microbiota and grapevine will help to develop sustainable strategies for grapevine protection.

## 1. Introduction

The concept of commercial grapevine nurseries, where grafted plants are propagated to be sold to growers, is something that has been developed largely in Europe since the late 19th century with the introduction of the North American aphid *Phylloxera* [1]. During the 1980s, grapevine propagation was modernized by the introduction of rapid machine-grafting procedures, in particular omega bench grafting [2]. Grapevines are relatively easy to propagate, but the process involves numerous steps with high organization and skill requirements to produce millions of vines of high quality [3]. Dormant cuttings are taken from rootstock and scion mother vines for bench grafting, rooting, or field budding. Nursery practices include cold storage, disbudding and hydration of rootstock/scion cuttings, grafting, and callusing and rooting of grafted plants [4]. A precise description of the stages and practices for the production of grafted plants was reviewed by Gramaje and Armengol [4].

The grapevine is considered an excellent model plant system for research on fungal and bacterial microbiota. Novel high-throughput sequencing (HTS) approaches have been recently used to outline the microbiome in grapevine organs such as roots, berries and leaves in mature vines due of its importance in grape production, fruit and foliar diseases management, and the effect of endemic microorganisms on the local characteristic of a wine [5,6,7]. Culture-dependent microbial approaches have historically been used to reveal microbiota present in the grapevine endospheres [8,9,10,11]. However, culture-independent high-throughput amplicon sequencing (HTAS) techniques have recently been deployed to increase the microbiome portrait of grapevine woody organs such as the trunk and cane [12,13,14,15,16,17,18].

While most of the abovementioned research has focused on scions and rootstocks at the vineyard level, little is known about the microbial composition of rootstocks in nurseries and how their composition changes following planting. Rootstocks are the link between soil and scion in grafted woody crops and have played a fundamental role in viticulture since the introduction of the aphid *Phylloxera*. Numerous studies have demonstrated the significant effect that grapevine rootstocks have on the scion performance. Rootstocks can provide tolerance to abiotic and biotic stresses, and they are also a major determinant of grapevine vigor and, consequently, of yield and grape quality [2]. The vascular system of grapevine rootstocks is still an unexplored niche despite its potential for hosting pathogenic and beneficial microorganisms. For instance, the presence of endogenous pathogens, such as trunk disease fungi, in grapevine rootstock planting material in newly established vineyards has been identified as a cause of yield losses, poor vine vigor, and long-term economic losses to the industry [19]. Some of the most common pathogens able to infect rootstock planting material in grapevine nurseries include fungi associated with black-foot and Petri diseases [4]. Up to 28 *Cylindocarpon*-like asexual morphs have been reported to cause black-foot disease [20,21]. Vines affected by black-foot show necrotic lesions on roots and black discoloration at the base of the rootstock [20]. Petri disease is caused by several ascomycetous fungi, including *Phaeoacremonium* spp., *Phaeomoniella chlamydospora* and *Cadophora luteo-olivacea*. Cross-sections of Petri disease-affected wood reveal black spots in the xylem vessels and black to brown vascular streaking. Potential inoculum sources of these fungi in grapevine nurseries include mother blocks, hydration tanks, grafting machines, callusing rooms, and nursery fields [4].

Plant-associated microbiomes are diverse and complex. There is still a limited understanding of the mechanisms and factors that establish and maintain specific plant-associated microbial communities, and the factors that stimulate the appropriate balance of different microbes. A better understanding of the microbiota-plant interaction during the early stages of the grapevine propagation process would help enhance applications that promote protection from pathogens and grapevine growth. The dynamics of a single fungal community over time can reveal more detail about community member interactions than a one-time snapshot from different communities in similar niches. To date, the temporal dynamics of the fungal microbiome occurring in propagating material have not been studied by HTAS, and available data are only referred in the context of culture-dependent approaches at specific stages of the production of vines [11,22,23].

We tested the following hypotheses: (1) the composition and diversity of fungal microbiome that inhabits the vascular system of grapevine rootstocks changes according to the practice in the propagation process; (2) fungal pathogen abundances are enhanced after specific procedures in the propagation process; (3) nursery practices affect the metabolic function of the fungal communities, and (4) some GTD pathogens are primary invaders of the grapevine rootstock vascular systems and can interact each other during the propagation process. The objective was to investigate the changes in the composition of fungal communities at different stages of the grapevine propagation process by HTAS.

## 2. Materials and Methods

### 2.1. Planting Material

Dormant grapevine cuttings of rootstocks 110 Richter (110 R) and 41 Berlandieri (41 B) were obtained from commercial nursery mother fields in Logroño (northern Spain). Two stocks of 15 cuttings per rootstock were used. Each stock was collected in different mother fields separated by 800 m. Rootstock mother vines were 12 years old and were cultivated along the ground from a self-supporting crown approximately 40 cm above the soil surface. Within each mother field, the 15 cuttings were randomly collected from 15 plants (one cutting per plant) near the crown of the mother vine. All rootstocks cuttings were 40 cm long and 1.5 cm in diameter. Data from each rootstock were analyzed independently due to the previously reported grapevine rootstock genetic control of the microbiome [24], and the variable degree of susceptibility of each grapevine rootstock to fungal trunk pathogen infections [25,26].

### 2.2. Wood Sample Collection

Planting material followed the fundamentals of the standard grapevine propagation process described by Gramaje and Armengol [4]. Cuttings were collected from rootstock mother plants in December 2017 and brought immediately to the laboratory for sampling (sampling time 1). A non-destructive method based in a 0.5 mm micro drill MICROMOT 50/EF (Proxxon micromot, Madrid, Spain) was used to collect grapevine wood from the xylem vessels of 110 R and 41 B rootstocks [27]. Woody tissues were collected from three points: the base (1 cm above the bottom part of the cutting), mid-point and apical (1 cm below the top of the cutting) part of each rootstock cutting. In each plant point, bark was first disinfected with 70% ethanol, a 1 cm^2^ flap was opened with a sterile scalpel, and 50 mg of woody tissue was collected in sterile Eppendorf tubes. Tissue from the three plant parts was mixed for DNA extraction. Sampling holes were covered with Parafilm after drilling [27].

Cuttings were then returned to the commercial nursery and held in cold storage at 2 °C with 90% humidity until March 2018. Following cold storage, rootstock cuttings were soaked in water for 24 h. After hydration, woody tissues were collected from the same plant parts following the same procedure as previously described (sampling time 2). Rootstock cuttings were then returned to the commercial nursery and bench-grafted with Tempranillo clone 1033 scion cuttings using an omega-grafting machine. Scion cuttings were randomly collected from a single mother field near the rootstock mother blocks. Following grafting, the graft unions were dipped in a melted wax formulation to encouraged graft union callus development. Grafted plants were packed in boxes with sterile water and placed in a callusing room at 26 °C and 80% humidity for 20 days until callus formed at the basal part of the plant and around the graft union. Following successful callusing, grafts were removed from the callusing boxes and woody tissues were collected again following the same procedure as previously described. The apical part consisted of rootstock wood collected 1 cm below the graft union (sampling time 3). Grafted vines were then transported and planted in an open-root field nursery in May 2018 with an in-plant spacing of 10 cm. The vines followed a regular program of drip irrigation and weed control. Dormant field-finished plants were lifted in December 2018 by hand. Woody tissues were collected from the same rootstock parts following the same procedure as previously described (sampling time 4). No biocontrol agents or chemicals were applied during the different stages of the propagation process.

### 2.3. DNA Extraction, Sequencing and Data Analysis of the High-Throughput Amplification Assay

DNA was extracted from the xylem tissue collected at each sampling time using the i-genomic Plant DNA Extraction Mini Kit (Intron Biotechnology, Seongnam-si, Korea). Quantification of DNA yields from each sample was performed by the Invitrogen Qubit 4 Fluorometer with Qubit dsDNA HS Assay (Thermo Fisher Scientific, Waltham, MA, USA), and the extracts were adjusted to 10 ng/μL. Samples of each rootstock (two stocks of 15 cuttings/grafted plants: 30 cuttings/grafted plants per rootstock) were then pooled in groups of three, resulting in a total of ten replicates for each rootstock. A total of 80 DNA samples (10 replicates × 4 times: 40 DNA samples per rootstock) was analyzed. The primers ITS86F (5′ GTGAATCATCGAATCTTTGAA 3′) [28] and ITS4 (5′ TCCTCCGCTTATTGATATGC 3′) were used to amplify the complete fungal ITS2 region (around 300 bp) [29]. Illumina sequencing primer sequences were attached to their 5′ ends.

PCRs were carried out in a final volume of 25 µL containing 12.5 µL of Supreme NZYTaq 2 × Green Master Mix (NZYTech, Lisboa, Portugal), 0.5 µM of the primers, 2.5 µL of template DNA and ultrapure water up to 25 µL. The following PCR protocol was used: initial denaturation at 95 °C for 5 min, followed by 35 cycles of 95 °C for 30 s, 49 °C for 30 s, 72 °C for 30 s, and a final extension step at 72 °C for 10 min. In a second PCR round, the oligonucleotide indices were attached with identical conditions. However, only five cycles and 60 °C as the annealing temperature were used for a schematic overview of the library preparation process. Contamination during library preparation in every PCR round was checked by including a negative control that contained no DNA. A positive control consisting of DNA from a grapevine wood sample previously evaluated by ITS2 HTAS was also included [18]. Library size was verified in 2% agarose gels stained with GreenSafe (NZYTech, Lisboa, Portugal). Libraries were purified using the Mag-Bind RXNPure Plus magnetic beads (Omega Biotek, Norcross, GA, USA), and then pooled in equimolar amounts according to the quantification data provided by the Qubit dsDNA HS Assay (Thermo Fisher Scientific, Waltham, MA, USA). The pool was sequenced in a MiSeq PE300 run (Illumina, San Diego, CA, USA). Control samples were sequenced to evaluate potential contaminations of the process.

Data analysis was done as described by Martínez-Diz et al. [18] using clustering in SCATA (https://scata.mykopat.slu.se/, accessed on: 29 November 2020). The OTU table, metadata and taxonomic classifications used in this study have been deposited in figshare (ID: 125710). HTAS data have been deposited in GenBank/NCBI under BioProject Acc. No. PRJNA776141.

### 2.4. Fungal Diversity, Taxonomy Distribution and Statistical Analysis

Alpha-diversity was calculated by analyzing the Chao1 richness and Shannon diversity in Phyloseq package. Differences in fungal alpha-diversity among stocks and nursery stages were inferred by multiple mean comparisons using Tukey’s honestly significant difference range test (*p* ≤ 0.05). PERMANOVA was used to infer which OTUs significantly differed in relative abundance among experimental factors after Bonferroni corrections. The relationship in OTUs composition among samples was investigated by calculating Bray Curtis metrics and visualized in PCoA plots. Good’s coverage values and rarefaction curves were also calculated. All diversity analyses were made using MicrobiomeAnalyst [30]. Persistent and transient microbiota were inferred using TIME [31]. Persistent fungal microbiota was defined as those taxa observed in 20% or more of the sampling times but with at least 90% of those observations being consecutive [32]. Transient fungal microbiota were defined as those taxa observed in at least 60% of the samples, but with at most 75% of those observations being consecutive (stages of sample development) [32].

The identification of fungal taxa that differed in relative abundance among sampling times was performed by computing the Linear Discriminant Analysis Effect Size (LEfSe) algorithm in MicrobiomeAnalyst. The Linear Discriminant Analysis (LDA) threshold score was set up at 1.0 and Wilcoxon *p*-value at 0.05. The results are displayed in a dot plot. The fungal OTUs shared among sampling times were visualized by Venn-diagram analysis (https://bioinformatics.psb.ugent.be/webtools/Venn/, accessed on: 13 January 2021). Correlation networks were computed with the SparCC algorithm to identify potential interactions between fungal genera that could represent parasitic, commensal, mutualistic or competitive relationships, using MicrobiomeAnalyst. *p*-value threshold was set up at 0.05 with 120 permutations, and the correlation threshold at 0.6.

Heatmaps were employed to visualize the abundances of GTD fungi at each sampling time using MicrobiomeAnalyst, with Euclidean as distance measure and Ward as a clustering algorithm. An ANOVA with log transforms was performed to compare the percentage abundance of each fungal genus associated with GTDs among sampling times. Normality of residuals was checked by Shapiro-Wilk’s test, and homogeneity of the variance by Levene’s test. Means were compared using Tukey’s test (*p* ≤ 0.05).

### 2.5. Functional Prediction of Fungal Communities

The function of fungal communities in the four sampling times in both rootstocks was assessed using FUNGuild v1.0 [33]. Three trophic modes were considered, saprotrophs pathotrophs and symbiotrophs. A total of eight guilds were classified within each trophic mode: ectomycorrhizal fungi, lichenized fungi, fungal endophytes, wood saprotrophs, dung saprotrophs, soil saprotrophs, undefines saprotrophs and plant pathogens. A fungal database was used to assign three confidence ranks, namely “highly probable”, “probable”, and “possible”. An “Unassigned” rank was used for OTUs that did not match taxa in the database. The effect of sampling times on the relative abundance of OTUs was assessed by ANOVA using Statistix 10 software (Analytical Software). Tukey’s test was used to compare transformed data means (*p* = 0.05).

### 2.6. Statistical Analysis

Differences in HTAS abundance were determined between sampling times using a one-way ANOVA test using Statistix 10 software. Tukey’s honestly significant difference test was used to compare data means (*p* = 0.05).

## 3. Results

### 3.1. Sequencing Depth and Community Diversity

After paired-end alignments, quality filtering and deletion of chimeras and singletons, a total of 8,665,871 fungal ITS2 sequences were generated from 78 samples (two samples were removed from the analysis due to the low number of reads) and assigned to 376 fungal operational taxonomic units (OTUs) (Supplementary Materials Table S1).

Nine fungal genera were identified in the negative control. These sequences were removed from the abundance of that OTU in the experimental samples [34]. According to the Good’s coverage values, 96.9% of total species richness was accounted for in fungal communities (Supplementary Materials Table S2). All diversity was captured with an adequate sequencing depth (Supplementary Materials Figure S1). The Chao1 richness estimator ranged from 27 to 59.5, and the Shannon diversity estimator ranged from 1.32 to 3.05 (Supplementary Materials Table S2).

### 3.2. Effect of Nursery Stages on Diversity and Community Membership

**Figure 1:**
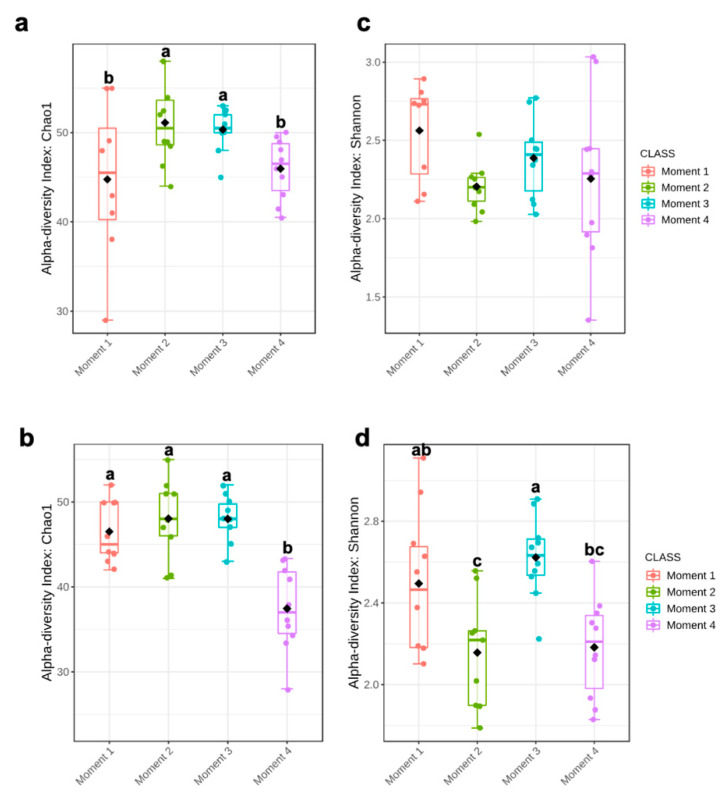
Boxplot illustrating the differences in (**a**) Chao1 and (**c**) Shannon diversity measures in 110 R rootstock, and (**b**) Chao1 and (**d**) Shannon diversity measures in 41 B rootstock of the fungal communities in four sampling moments of the propagation process in grapevine nurseries: before cold storage (Moment 1), after hydration (Moment 2), after callusing (Moment 3), and after rooting in field nurseries (Moment 4). Alpha-diversity differences were compared using one-way ANOVA with Tukey’s test. *p* > 0.05. Means followed by the same letter do not differ significantly.

**Figure 2:**
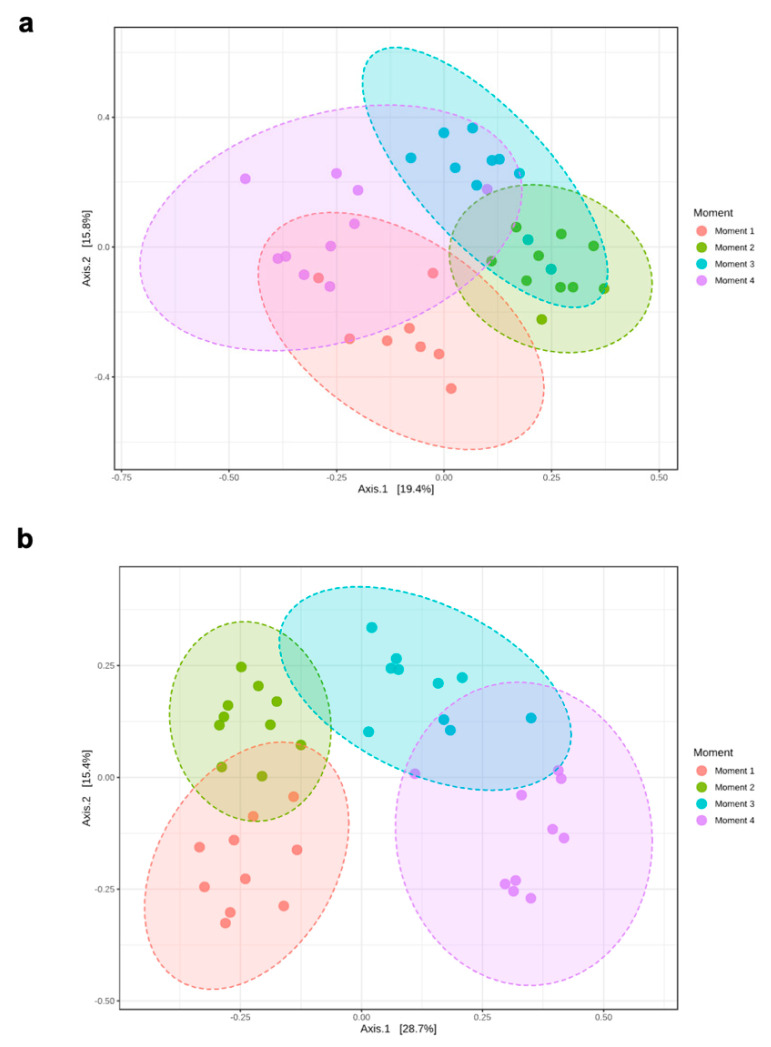
Principal Coordinate Analysis (PCoA) based on Bray–Curtis dissimilarity metrics showing the distance in the fungal communities among sampling moments in (**a**) 110 R (**b**) 41 B rootstocks.

Alpha-diversity of fungal communities in both grapevine rootstocks wood samples did not differ significantly between plant stocks (Table 1); thus, the data of both stocks for each rootstock were combined for analyses. In general, the alpha-diversity of fungal communities differed among sampling times for both rootstocks (*p* < 0.05) (Table 1; Figure 1). Sampling time did not predict Shannon diversity in 110 R rootstock (Table 1). In 110 R, taxa richness (Chao1) was lower in sampling times 1 and 4 compared to sampling times 2 and 3 (Figure 1). In 41 B, taxa richness was lower in sampling time 4 compared to the other sampling times, while taxa richness and evenness (Shannon) provided the lowest values for sampling times 2 and 4, although no significant differences were found between sampling times 1 and 4 (Figure 1). Principal coordinates analysis (PCoA) of Bray Curtis data demonstrated that sampling time was the primary source of beta-diversity in both 110 R (*R*^2^ = 0.39, *p* < 0.001) and 41 B (*R*^2^ = 0.56, *p* < 0.001) rootstocks (Figure 2).

**Figure 3:**
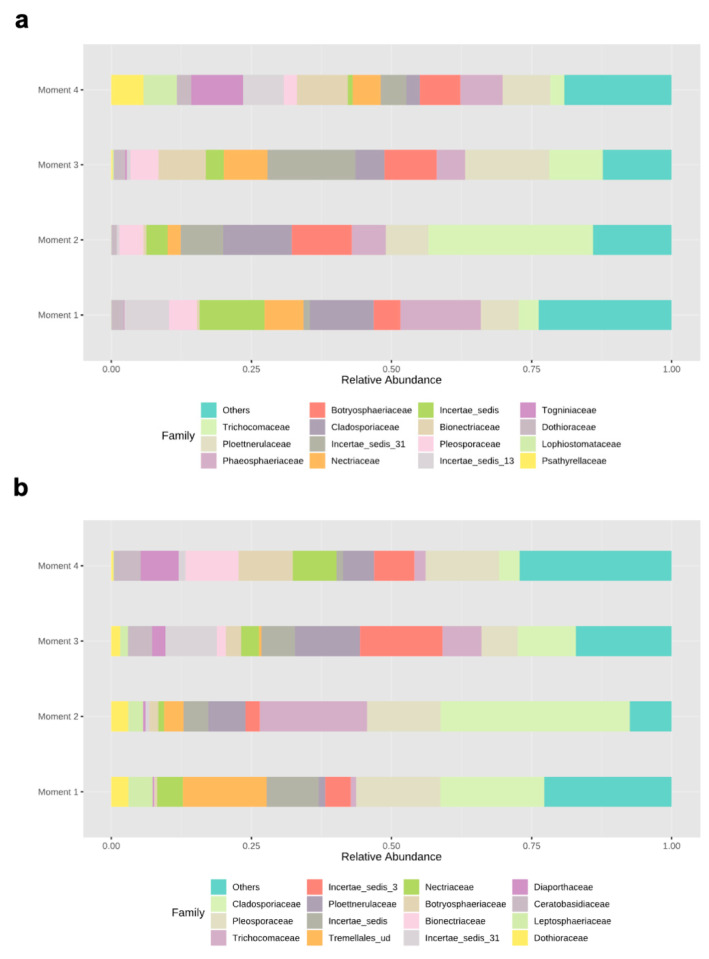
Relative abundances of different fungal families in (**a**) 110 R and (**b**) 41 B rootstocks.

Ascomycota dominated the fungal phyla across sampling times in both rootstocks, with percentages of abundances ranging from 86% (sampling time 1) to 98% (sampling time 2) in 110 R, and from 76% (sampling time 1) to 93% (sampling time 4) in 41 B (Supplementary Materials Figure S2). In the 110 R rootstock, the lowest values of fungal richness and diversity were obtained at sampling times 1 (46 ± 9.1) and 3 (2.2 ± 0.3), respectively (Figure 1). The most abundant families were Phaeosphaeriaceae (14.3%) and Cladosporiaceae (11.3%) at sampling time 1, Trichocomaceae (29.4%) and Cladosporiaceae (12.2%) at sampling time 2, Incertae sedis 31 (15.6%) and Ploettnerulaceae (15.1%) at sampling time 3, and Togniniaceae (9.1%) and Bionectriaceae (9.0%) at sampling time 4 (Figure 3). The core communities were dominated by Cladosporiaceae, Dothioraceae, Nectriaceae, Pleosporaceae, Ploettnerulaceae and Trichocomaceae. The persistent community was composed of an unknown family within the Sordariales and Incertae sedis 13 family, while the transient community was composed of Bulleribasidiaceae, Ceratobasidicaceae, Filobasidiaceae, Helotiaceae, Herpotrichiellaceae, Schizophyllaceae, Sclerotiniaceae and Xylariaceae. Seven (2.4%) and twenty (6.8%) genera were defined as persistent and transient taxa, respectively (Supplementary Materials Table S3).

In the 41 B rootstock, the lowest value of fungal richness was obtained at sampling time 4 (37 ± 7.3), while the lowest values of fungal diversity were obtained at sampling times 2 (2.2 ± 0.4) and 4 (2.2 ± 0.3), respectively (Figure 1). The most abundant families were Cladosporiaceae (18.5%) and Pleosporaceae (15.0%) at sampling time 1, Cladosporiaceae (33.7%) and Trichocomaceae (19.1%) at sampling time 2, Incertae_sedis_3 (14.7%) and Ploettnerulaceae (11.6%) at sampling time 3, and Pleosporaceae (13.1%) and Botryosphaeriaceae (9.7%) at sampling time 4 (Figure 3). The core communities were dominated by Botrosphaeriaceae, Cladosporiaceae, Dothioraceae, Incertae sedis 3 and 13, Leptosphaeriaceae, Nectriaceae, Phaeosphaeriaceae, Pleosporaceae, Ploettnerulaceae and Trichocomaceae families. The persistent community was composed of unknown families within the orders Tremellalles and Sordariales, Bulleribasidiaceae, Schizophyllaceae and Trichosporonaceae families. The transient community was composed of Chaetothyriaceae, Helotiaceae, Incertae sedis 25 and 31, Lophiostomataceae, Sarocladiaceae and Xylariaceae families. Nine (3.3%) and fourteen (5.2%) genera were defined as persistent and transient taxa, respectively (Supplementary Materials Table S3).

### 3.3. Specific and Shared Fungal Assemblages

The percentage of shared fungal OTUs among the four sampling times were similar in both rootstocks: 24.8% (110 R) and 21.4% (41 B) (Supplementary Materials Figure S3). Core taxa with more than 50% prevalence were composed by *Cadophora* (94.6%), *Cladosporium* (86.6%), *Penicillium* (78.3%), *Eucasphaeria* (72.9%), *Paraphoma* (64.8%), *Fusarium* (64.8%) and *Alternaria* (56.7%) in 110 R rootstock, and *Alternaria* (94.8%), *Cladosporium* (89.7%), *Acremonium* (71.8%), *Cadophora* (61.5%), *Penicillium* (53.8%) and *Aureobasidium* (51.2%) in the 41 B rootstock (Supplementary Materials Figure S4). Specific OTUs associated with each sampling time ranged from 6.8 to 14.6% (110 R), and from 8.1 to 15.5% (41 B). The OTUs that were unique in each sampling time for each rootstock are shown in Supplementary Materials Table S4. Four, one and fifteen unique taxa were identified in both rootstocks at sampling times 1, 3 and 4, respectively. No unique fungal taxa were present in both rootstocks at sampling time 2 (Supplementary Materials Table S4).

**Figure 4:**
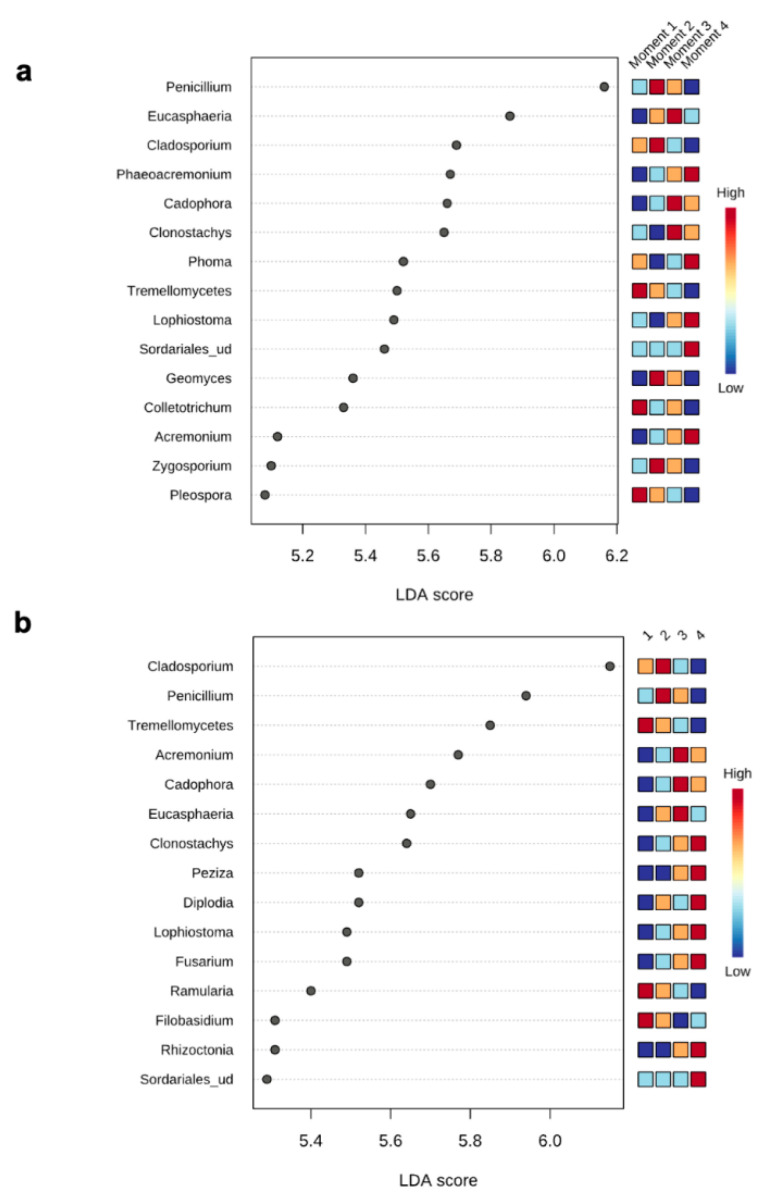
LEfSe analysis showing the genera with significant differential abundances in each sampling moment for 110 R (**a**) and 41 B (**b**) rootstocks. The colors in the heatmap represent the abundances of genera.

**Figure 5:**
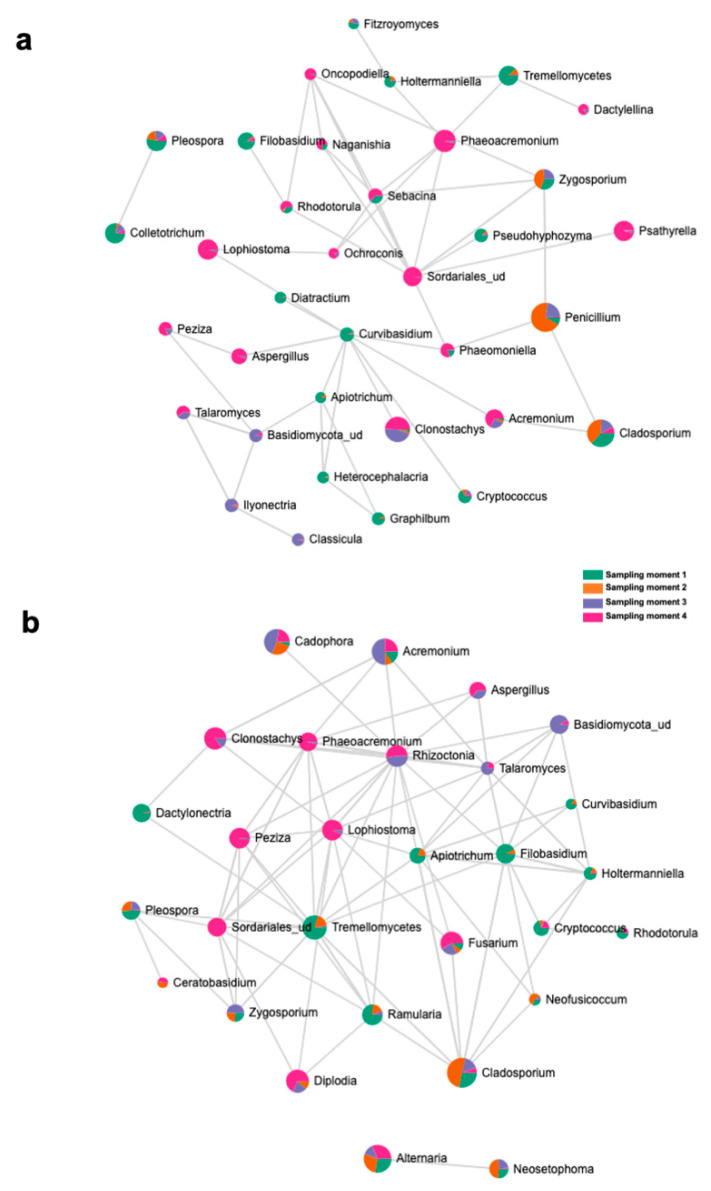
SparCC correlation analysis at the genus level among sampling moments in (**a**) 110 R and (**b**) 41 B rootstocks.

The top 15 fungal clades in the grapevine internal tissues detected by the LEfSe analysis in 110 R and 41 B rootstocks are shown in Figure 4. Sampling time 4 showed higher number of differentially abundant fungal clades (five and seven in 110 R and 41 B, respectively). At sampling time 1, dominant fungal genus in both rootstocks was Tremellomycetes. At sampling time 2, dominant fungal genera were *Penicillium* (110 R) and *Cladosporium* (41 B). At sampling time 3, dominant fungal genera were *Eucasphaeria* (110 R) and *Acremonium* (41 B), while *Phaeoacremonium* (110 R) and *Clonostachys* (41 B) represented the dominant fungal genera at sampling time 4 (Figure 4). In 110 R rootstock, 49 interactions were identified between fungal taxa, 34 positive and 15 negative (Figure 5; Supplementary Materials Table S5). *Curvibasidium* and an unknown genus within the Sordariales established the highest number of correlations (*n* = 9) with other fungal taxa. In the 41 B rootstock, 80 interactions were identified between fungal taxa, 54 positive and 26 negative (Figure 5; Supplementary Materials Table S6). *Rhizoctonia* had the highest number of correlations (n = 15) with other fungal taxa.

### 3.4. Nursery Propagation Stages Affect Fungal Functionality

**Figure 6:**
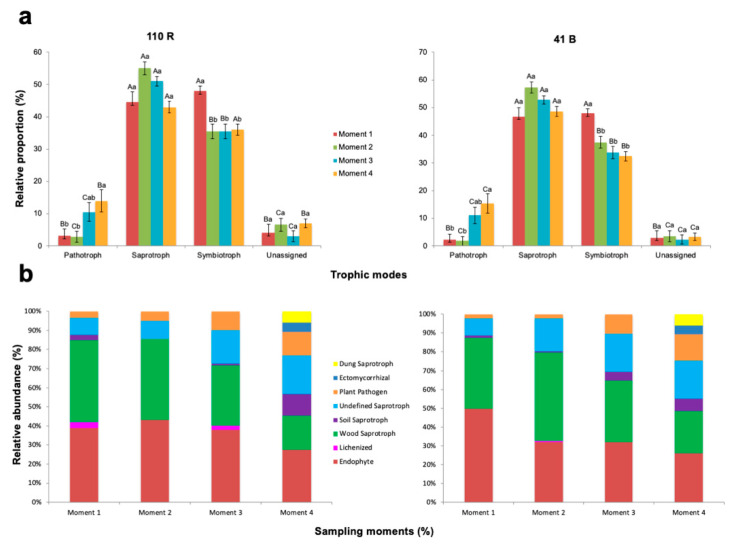
Variations in (**a**) fungal function and (**b**) composition of fungal functional groups (guilds) inferred by FUNGuild. Tukey’s test at *p* > 0.05 level. Means followed by the same letter do not differ significantly (*p* > 0.05). Capital letters are for comparison of means among functional groups within each sampling moment. Small letters are for comparison of means among sampling moments within each functional group.

There were significant differences in the relative proportion of fungal functions within each sampling time for both rootstocks (*p* < 0.05) (Figure 6; Supplementary Materials Table S7). The trophic mode at sampling time 1 was dominated by symbiotrophs which accounted for 48.0% and 47.9% of the total OTUs in 110 R and 41 B rootstocks respectively, but saprotrophs were not significantly different (*p* < 0.05). At the remaining sampling times, saprotrophs were found in higher proportions compared to the other groups (Figure 6; Supplementary Materials Table S7). The relative proportion of pathotrophs was higher in sampling time 4 (14.1% in 110 R and 15.4% in 41 B) and lower at sampling time 2 (2.9% in 110 R and 1.8% in 41 B) for both rootstocks.

The relative abundances of endophytes were higher at sampling time 2 in 110 R (43.1%) and at sampling time 1 in 41 B (49.8%), and lower at sampling time 4 in both rootstocks (27.4 and 26.1%, respectively) (Supplementary Materials Table S8). The relative abundances of wood saprotrophs were higher at sampling times 1 (43%) and 2 (42.5%) in 110 R, and at sampling time 2 in 41 B (47.1%), and lower at sampling time 4 in both rootstocks (18.0 and 22.2%, respectively). The relative abundances of soil saprotrophs at sampling time 4 in 110 R (11.4%), and at sampling times 3 (4.7%) and 4 (6.7%) in 41 B were significantly higher than in the other sampling times (*p* < 0.05) (Supplementary Materials Table S8). Undefined saprotrophs were significantly less abundant at sampling times 1 (9.0%) and 2 (9.5%) in 110 R, and in sampling time 1 (9.1%) in 41 B (*p* < 0.05). Plant pathogens were detected in higher abundances at sampling time 4 in both rootstocks (12.5% in 110 R and 14.1% in 41 B), with no significant differences at sampling time 3 (*p* > 0.05). Lichenized fungi were only found at sampling times 1 (3%) and 3 (2%) in 110 R, and at sampling time 2 in 41 B (0.4%). In both rootstocks, ectomycorrhizal and dung saprotrophs were only identified at sampling time 4 (Supplementary Materials Table S8).

### 3.5. Shifts in Fungal Trunk Pathogen Infections through the Propagation Process

**Figure 7:**
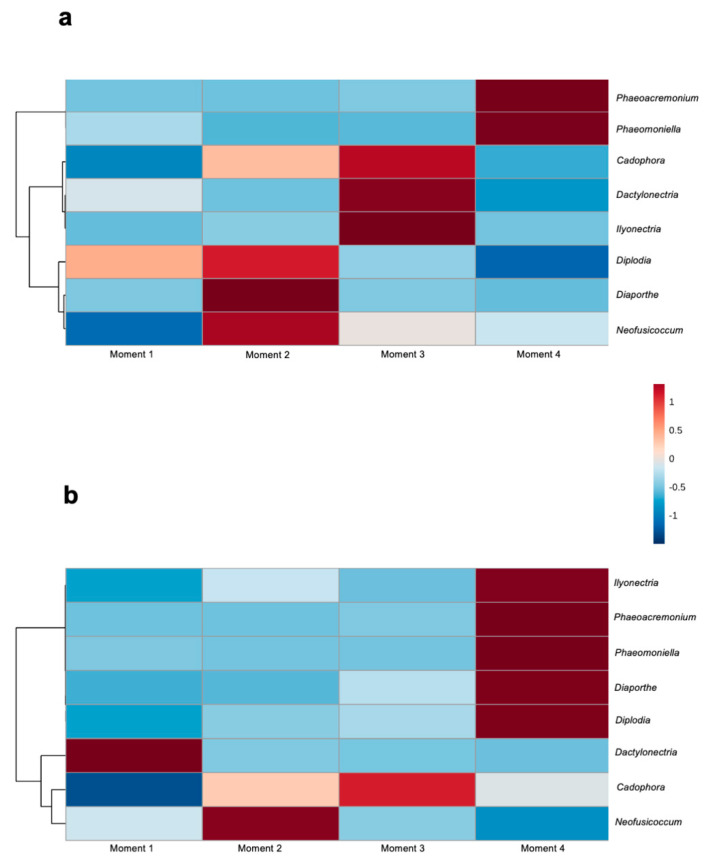
Hierarchical clustering heat map of grapevine trunk diseases associated genera from (**a**) 110 R and (**b**) 41 B rootstocks in each sampling moment using Euclidean distance measure and Ward clustering algorithm.

Among the identified fungal taxa in both rootstocks, eight genera have previously been associated with GTDs: Cadophora, Dactylonectria, Diaporthe, Diplodia, Ilyonectria, Neofusicoccum, Phaeoacremonium and Phaeomoniella. In both rootstocks, the abundances of the Petri disease pathogens Phaeoacremonium and Phaeomoniella were higher at sampling time 4, while the abundances of Cadophora and Neofusicoccum were higher at sampling times 3 and 2, respectively (Figure 7). In 110 R, the abundances of the black-foot pathogens Ilyonectria and Dactylonectria were higher at sampling time 2, while the abundances of Diplodia and Diaporthe were higher at sampling time 2. In 41 B, the abundances of Ilyonectria, Diaporthe and Diplodia were higher at sampling time 4, while the abundance of Dactyonectria was higher at sampling time 1 (Figure 7).

## 4. Discussion

In this study, we examined the temporal dynamics of the fungal microbiome in grapevine rootstocks through four stages of the propagation process using a non-destructive method. To date, research on grapevine microbiomes have predominantly focused on the scion cultivar, as it is the visible half-part of the vine and produces the fruit. However, more than 80% of the vineyards worldwide are currently grafted onto rootstocks [35], which have a significant influence on crop yield, grape quality and give protection against pathogens and pests [2].

The fungal microbiome in both rootstocks analysed (110 R and 41 B), was dominated by Ascomycota throughout the propagation process. This result is consistent with previous HTAS studies that explored grapevine endophytic fungal communities [14,15,16,17,18,36,37,38,39,40,41,42,43].

A total of 376 OTUs were detected in this study, ranging from 154 to 172 OTUs, and from 241 to 250 OTUs, in 110 R and 41 B rootstocks, respectively. The high number of OTUs found in this study differs from culture-dependent approaches. In Switzerland, Casieri et al. [44] identified only 66 OTUs occurring in the wood of 1-year-old *V. vinifera* grafted plants, whereas Hofstetter et al. [45] isolated 85 fungal species from healthy nursery planting material. In France, Bruez et al. [46] identified 48 OTUs from healthy wood tissues of the trunk disease esca leaf-asymptomatic and symptomatic vines. Similarly, Kraus et al. [11] identified 86 OTUs in healthy grapevine branches of different ages (from 2-month to 8-year-old) in Germany. It should be noted however that the OTU accumulation curves produced for each branch age hardly started to saturate, suggesting that the number of OTUs would probably increase with a larger number of samples. Furthermore, a comparative study [15] that investigated changes in the potentially active fungal communities of internal grapevine wood after hot-water treatment (HWT) in nursery material revealed that a HTAS-based procedure was superior to traditional isolation for the detection and identification of fungal communities.

In this study, the core microbiome was composed of the genera *Cadophora*, *Cladosporium*, *Penicillium* and *Alternaria* in both rootstocks, together with *Eucasphaeria*, *Paraphoma* and *Fusarium* in 110 R, and *Acremonium* and *Aureobasidium* in 41 B. These results are in line with recent studies [11,18,36,37] focused on exploring the interior of grapevine wood. The ubiquitous, fast-growing fungi *Cladosporium*, *Alternaria* and *Aureobasidium* were previously found to dominate the fungal communities in the xylem vessels of healthy grapevine branches of all ages in Germany [11]. They were also the dominant fungal taxa in California [36]. In addition, these fungal genera were frequently found colonizing the grapevine wood after pruning in Spain [18], and *Cladosporium* as the dominant fungal taxon inhabiting several biocompartments of the grapevine endosphere in California [37]. The fungal genera *Acremonium*, *Fusarium* and *Penicillium* were also prevalent in grapevine nursery plants, even after HWT, in Spain and Czech Republic [15]. Regarding *Eucasphaeria,* fungal species belonging to this genus were isolated from grapevine nursey plants in Switzerland [45] and, interestingly, the species *E. capensis* was isolated from a zone of dark discoloured wood in a rootstock in Germany, although its pathogenicity in grapevine wood is unknown [47]. Recently, *Eucasphaeria* was found colonizing grapevine pruning wounds in Spain [18]. Fungi belonging to *Paraphoma* genus, a *Phoma*-like fungi [48], were previously found inhabiting both esca-symptomatic and asymptomatic vines, as well as grapevine nursery plants in Switzerland [45]. The role of *Aureobasidium,* in particular the species *A. pullulans,* as a potential biocontrol agent of fungal trunk pathogens of grapevine has been demonstrated in vitro [49] and *in planta* [50]. Species of this genus have been shown to prevail in the core microbiome of grapevines in recent studies [14,18,36,38]. The presence of *Cadophora* spp. has been associated with Petri disease young vines and esca in mature vines, and has been found in the woody tissue of nursery stock [15,22,45,50,51,52] at all stages of the propagation process in nurseries [23], and in mature vines [11,14,18,20].

The fungal genus *Neofusicoccum* was found as a persistent taxon in both rootstocks, which confirms this genus as a primary settler of grapevine vascular tissues. This genus belongs to the Botryosphaeriaceae family and is considered one of the most virulent fungal genera associated with the trunk disease Botryosphaeria dieback [53,54], *Neofusicoccum parvum* being the most common *Neofusicoccum* species isolated from grapevine worldwide [55]. The role of rootstock mother vines as a primary source of *Neofusicoccum* spp. has been well-documented by Aroca et al. [28]. *Neofusicoccum* spp. are spread by the dispersion of airborne spores that penetrate the mother plants and mature vines through pruning wounds [20]. Previous research has found *Neofusicoccum* spp. inhabiting rootstocks from nursery material at different stages of the propagation process [15,51,55,56].

In this study, fungal taxa richness and diversity generally decreased through the propagation process. This result was surprising since we expected an increase of fungal diversity after root development stage in the field nursery where multiple interactions between the plant and soil microorganisms can occur. This could be partially attributed to the enhancement of microbial interactions with planting material in nursery practices, such a hydration and callusing. For instance, hydration has been identified as a potential source of cross contamination by microorganisms [57].

Fungal functionality analysis showed that the relative abundance of endophytes decreased throughout the propagation process, while the abundance of plant pathogens increased towards the last stage and before selling the plant to the grower. We identified eight genera associated with GTDs. Among these, the relative abundance of genera associated with black-foot, such as *Ilyonectria* in 110 R, and genera associated with Petri disease, such as *Phaeomoniella* and *Phaeoacremonium,* in both rootstocks increased significantly after root development in the nursery field. The soilborne black-foot pathogens are commonly found in nursery field soils [58], and their capacity to infect grafted grapevine once planted in the field nursery is well documented [59,60]. A survey carried out in Spanish nurseries highlighted the relatively higher abundance of black-foot fungi after one growing season in field nurseries compared to their abundances in hydration tanks and callusing rooms [61]. Petri disease pathogens can spend part of their life cycle in vineyard soils, which allows them to infect young vines through the roots [61]. Wounds made during the nursery process can also be an important point of entry for GTD fungi, including Petri disease pathogens [4] or fungi associated with Botryosphaeria [62] and Phomopsis [28] diebacks. This is in line with the results of our study, which detected high levels of GTD fungi such as *Neofusicoccum* spp. (Botryosphaeria dieback) and *Diaporthe* spp. (Phomopsis dieback) in 110 R, and *Neofusicoccum* spp. in 41 B, during the early stage of the propagation process. Surprisingly, correlation network analysis resulted in low levels of connectivity among GTD fungi at all stages of the propagation process, even though co-infections among these pathogens are common in vascular tissues of young vines [63,64].

## 5. Conclusions

A non-destructive method based on a micro drill was used to explore the temporal dynamics of the fungal microbiome in grapevine rootstocks through the propagation process in nurseries. Fungal communities in the vascular system of 110 R and 41 B grapevine rootstocks differed between the different stages of the propagation process in nurseries, the hydration stage being a potential source of cross contamination by trunk disease pathogens. Several genera associated with grapevine trunk diseases and/or potential biocontrol activity were identified in this study. The fungal pathogenic genus Neofusicoccum was identified as a primary settler of grapevine vascular tissues. Understanding the large diversity of fungi in the rootstock vascular tissue and the interactions between fungal microbiota and grapevine will help to develop sustainable strategies for grapevine protection.

## Figures and Tables

**Table 1:** Experimental factors predicting α-diversity of xylem associated fungal communities in 110 Richter and 41 Berlandieri rootstocks.

	110 Richter		41 Berlandieri	
	Chao1	Shannon	Chao1	Shannon
Stock	*F* = 0.15 *p* = 0.8702	*F* = 1.25 *p* = 0.2197	*F* = 0.58 *p* = 0.5612	*F* = 0.17 *p* = 0.8618
Sampling moment	*F* = 3.48 ***p* = 0.0261**	*F* = 1.95 *p* = 0.1393	*F* = 15.24 ***p* < 0.0001**	*F* = 6.89 ***p* < 0.0001**
Stock × sampling moment	*F* = 2.45	*F* = 3.21	*F* = 3.52	*F* = 2.88
	*p* = 0.1301	*p* = 0.2504	*p* = 0.1969	*p* = 0.1141

ANOVA, analysis of variance. All *p* values were corrected for multiple comparisons using the sequential Bonferroni correction. Bold values indicate statistically significant results after correction for multiple comparisons. *p* < 0.05.

## Data Availability

The OTU table, metadata and taxonomic classifications used in this study have been deposited in figshare (ID: 125710). HTAS data have been deposited in GenBank/NCBI under BioProject Acc. No. PRJNA776141.

## Supplementary Materials

The following supporting information can be downloaded at: https://www.mdpi.com/article/10.3390/jof8050421/s1. Figure S1. Rarefaction curve values for each sample in (a) 110 Richter and (b) 41 Berlandieri rootstocks. Figure S2. Relative abundance of different fungal phyla in (a) 110 Richter and (b) 41 Berlandieri rootstocks. Figure S3. Venn diagram illustrating the overlap of the number of OTUs identified in the fungal microbiota among sampling moments in (a) 110 Richter and (b) 41 Berlandieri rootstocks. Figure S4. Core microbiome analysis showing a limited number of genera prevalent across all the samples in (a) 110 R and (b) 41 B rootstocks. Sample prevalence threshold is set up above 20%, and relative abundance threshold is set up above 0.01%. The heatmap colors represent the sample prevalence values. Table S1. Number of reads, total OTUs, richness (Chao1 estimates of species richness) or diversity (Shannon’s index of diversity) indices expressed as average and standard deviation in the different steps of the nursery propagation process for both 110 R and 41 B grapevine rootstocks. Table S2. Estimates of sample coverage and diversity indices at the genus level for fungal profiles. Table S3. Persistent and transient fungal genera across sampling moments in each rootstock. Table S4. OTUs unique to each sampling moment. Table S5. SparCC correlation coefficients between taxa in 110 R rootstock. Table S6. SparCC correlation coefficients between taxa in 41 B rootstock. Table S7. Relative proportion (%) of fungal function from sampling moments inferred by FunGuild. Table S8. Compositions and relative abundance (%) of fungal functional groups (guild) inferred by FunGuild.
