# Supplementary material for: Exploring the Temporal Dynamics of the Fungal Microbiome in Rootstocks, the Lesser-Known Half of the Grapevine Crop
Source: J Fungi (Basel). 2022 Apr 20;8(5):421. doi: 10.3390/jof8050421 (PMC9144578; doi:10.3390/jof8050421)
Supplement: Supplementary file 1 [file jof-08-00421-s001.zip › jof-1669998-supplementary.pdf]

Supplementary Table S1. Number of reads, total OTUs, richness (Chao1 estimates of species richness) or diversity (Shannon's index of diversity) indices expressed as average and standard deviation in the different steps of the nursery propagation process for both 110 R and 41 B grapevine rootstocks.

**110 R**				
Index	Sampling moments[a]			
	1[b]	2	3	4
Reads	48,180±21638	231,740±135,430	105,848±39,321	94,057±36,243
OTUs	172	163	154	162
Chao1	46.7±8.1	51.3±6.3	49.6±2.3	46.1±2.9
Shannon	2.4±0.8	2.0±0.6	1.9±0.9	2.0±0.8
**41 B**				
Index	Sampling moments			
	1	2	3	4
Reads	65,898±26,511	110,078±41,661	95,126±42,618	123,516±70,218
OTUs	246	259	250	241
Chao1	46.5±3.6	48.3±4.1	48.2±2.5	37.0±7.0
Shannon	2.0±0.9	2.2±2.6	2.4±0.8	2.2±2.5

[a]Sampling moments: 1, before cold storage; 2, after hydration and before grafting; 3, after callusing, and 4, after rooting in nursery fields.

[b]Samples DG04 and DG07 were removed from the analysis due to the low number of sequences reads.

Supplementary Table S2. Estimates of sample coverage and diversity indices at the genus level for fungal profiles.

Sample ID	Good's coverage	Chao1 richness	Shannon diversity
DG01	1.000	27	2.09
DG02	0.991	45	2.63
DG03	0.991	52	2.71
DG05	0.994	43	2.74
DG06	0.988	47	2.24
DG08	1.000	34	2.11
DG09	0.933	43	2.61
DG10	0.921	38	2.82
DG11	0.995	35	2.54
DG12	1.000	50	2.64
DG13	0.933	41	2.17
DG14	0.933	53	2.79
DG15	0.999	45	2.18
DG16	0.987	44	2.08
DG17	0.931	42	2.15
DG18	0.991	49	3.05
DG19	0.923	47	2.55
DG20	0.962	41	2.34
DG21	0.987	40	2.04
DG22	0.995	55	2.21
DG23	0.892	52.5	2.26
DG24	0.900	45	1.97
DG25	0.903	50.2	2.48
DG26	0.987	54	2.26
DG27	0.995	40.5	2.16
DG28	0.987	51.5	2.18
DG29	0.927	43.2	2.08
DG30	0.987	50	2.27
DG31	0.994	47	2.23
DG32	0.991	44	2.01
DG33	0.903	41.4	1.77
DG34	1.000	49.5	2.61
DG35	1.000	44	1.87
DG36	0.995	38	1.89
DG37	0.981	50.5	2.28
DG38	0.988	59.5	2.21
DG39	0.981	49.5	2.48
DG40	0.981	49.5	2.35
DG41	0.909	41	2.28

DG42	1.000	45	2.44
DG43	1.000	41	2.63
DG44	0.909	43	2.33
DG45	0.999	42	2.36
DG46	0.987	44	2.01
DG47	0.995	51.7	2.05
DG48	0.995	45	2.39
DG49	0.994	44	2.71
DG50	0.895	51	2.08
DG51	0.958	43	2.85
DG52	0.910	47	2.53
DG53	0.958	49	2.55
DG54	0.958	47	2.83
DG55	0.958	47	2.75
DG56	0.920	47	2.41
DG57	0.962	45	2.22
DG58	0.958	44	2.68
DG59	0.900	44	2.53
DG60	0.958	40	2.63
DG61	0.999	43	2.31
DG62	0.855	39	2.84
DG63	0.995	42	1.92
DG64	0.999	37	2.24
DG65	0.999	40	2.34
DG66	0.981	44	1.97
DG67	0.988	37	1.71
DG68	0.981	44	2.19
DG69	0.981	42	2.92
DG70	0.960	37	1.32
DG71	0.995	37	2.38
DG72	0.995	33.5	2.28
DG73	0.960	37	1.87
DG74	0.995	24	1.83
DG75	1.000	36.5	2.04
DG76	0.995	33.6	1.93
DG77	1.000	43.3	2.10
DG78	1.000	44.2	2.34
DG79	0.995	42	2.29
DG80	0.995	38.7	2.58
Average	0.969	43.7	2.32

Supplementary Table S3. Persistent and transient fungal genera across sampling moments in each rootstock

110 R		41 B	
Persistent	Transient	Persistent	Transient
Neofusicoccum	*Pseudohyphozyma*	*Vishniacocyma*	*Saracladium*
Zygosporium	*Vishniacozyma*	*Neofusicoccum*	*Neosetophoma*
Pyrenochaeta	*Paraphoma*	*Unkwnon Sordariales genus*	*Periconia*
Phaeosphaeria	*Neosetophoma*	*Zygosporium*	*Stemphylium*
Unkwnon Sordariales genus	*Ramularia*	*Apiotrichum*	
Eucasphaeria	*Filobasidium*	*Tremellomycetes*	*Pyrenochaeta*
	Truncatella	*Clonostachys*	*Phaeosphaeria*
	Stemphylium	*Rhizoctonia*	*Anthostomella*
	Cryptococcus	*Ramularia*	*Eucasphaeria*
	Leucosporidium		*Aspergillus*
	Phoma		*Stagonosporopsis*
	Sporobolomyces		*Cyphellophora*
	Ophiosimulans		*Scytalidium*
	Leptospora		*Leptosphaeria*
	Anthostomella		*Pseudophiobolus*
	Stagonosporopsis		
	Cyphellophora		
	Chatophaeronema		
	Rhizoctonia		

Supplementary Table S4. OTUs that were unique to each sampling moment.

110 R			
Sampling moment 1	Sampling moment 2	Sampling moment 3	Sampling moment 4
Agaricus<br>*Amanita*<br>*Botryotinia**<br>*Calycina*<br>*Clavispora*<br>*Colacogloea*<br>*Cystobasidiomycetes**<br>*Diplosphaera*<br>*Dothideomycetes_ud*<br>*Hortaea*<br>*Lewia*<br>*Mrakia*<br>*Mrakiella*<br>*Mycena*<br>*Myrmecridium*<br>*Naohidea*<br>*Neodactylaria*<br>*Neodevrisea*<br>*Paraophibolus*<br>*Pleurocatena*<br>*Pleurotus*<br>*Powellomyces*<br>*Pseudoarthrographis*<br>*Pseudocoleophoma*<br>*Pseudoidymosphaeria*<br>*Ramophialophora*<br>*Rectipilus*<br>*Septoria*<br>*Spissiomyces*<br>*Taphrina**<br>*Terfezia*<br>*Udeniomyces**<br>*Valsa*<br>*Vertimonosporium*	*Achroceratosphaeria*<br>*Amycosphaerella*<br>*Boubovio*<br>*Conlarium*<br>*Crepidotus*<br>*Dendryphion*<br>*Didyma*<br>*Ijuhya*<br>*Kondoa*<br>*Leotiomycetes*<br>*Marasmius*<br>*Paraphaeospharia*<br>*Preussia*<br>*Pseudomicrostroma*<br>*Septoriella*<br>*Sporothrix*<br>*Subplenodomus*<br>*Thelebolus*<br>*Tricellula*<br>*Zymoseptoria*	*Acrophialophora*<br>*Arxiella*<br>*Calceomyces*<br>*Calloria*<br>*Chrysosporium*<br>*Clitopilus*<br>*Comoclathris*<br>*Coniosporium*<br>*Cordycipitaceae_ud*<br>*Cyanodermella*<br>*Cylindrobasidium*<br>*Dendrophoma*<br>*Fusariella*<br>*Kwoniella*<br>*Lasionectria*<br>*Mariannaea*<br>*Myriospora*<br>*Occultifur*<br>*Polysporus**<br>*Pseudodictyosporium*<br>*Sagenomella*<br>*Scopulariopsis*<br>*Simplicillium*<br>*Stephanonectria*<br>*Trichothecium*<br>*Zygomycete*	*Armillaria*<br>*Arthrobotrys**<br>*Cercophora**<br>*Coprinopsis**<br>*Cornuvesica*<br>*Crocicreas*<br>*Curvularia*<br>*Cylindrocarpon**<br>*Dendroclathra**<br>*Discostroma*<br>*Ermericella*<br>*Eutypa*<br>*Fusicolla*<br>*Glomeromycotina**<br>*Glomus**<br>*Hormonema*<br>*Humicola*<br>*Kalmusia*<br>*Keissleriella*<br>*Meira**<br>*Moesziomyces**<br>*Natantipora**<br>*Nectriaceae_ud*<br>*Nigrospora*<br>*Nothophoma*<br>*Paraconiothyrium*<br>*Paraglomus*<br>*Pestalotiopsis*<br>*Pithomyces*<br>*Pluteus*<br>*Puccinia**<br>*Pyxidiophora**<br>*Rhizophagus**<br>*Savoryella*<br>*Steomelanomma*<br>*Solicoccozyma**<br>*Sordariomyectes_ud*<br>*Stillbella*<br>*Tetrasphaeria*<br>*Tetracoccosporium*<br>*Variocosporellopsis**<br>*Zygosaccharomyces*
41 B			
Absidia<br>*Arthroderma*<br>*Bannoa*<br>*Botryotinia*<br>*Bulleromyces*<br>*Calloria*<br>*Chytridiales*<br>*Coenochlorosis*	*Anthopsis*<br>*Arhrinium*<br>*Beauveria*<br>*Biatriospora*<br>*Cenococcum*<br>*Cephalotrichum*<br>*Chrysosporium*<br>*Claussenomyces*	*Athelia*<br>*Crocicreas*<br>*Hydropisphaera*<br>*Lecanicillium*<br>*Leucosphaerina*<br>*Lophiotrema*<br>*Maireina*<br>*Monascrosporium*	*Albifimbria*<br>*Arthrobotrys*<br>*Cercophora*<br>*Coprinopsis*<br>*Cylindrobasidium*<br>*Cylindrocarpon*<br>*Dendroclathra*<br>*Erysiphe*

Coniosporium	*Debaryomyces*	*Nectriella*	*Exidia*
Cryptodiscus	*Dothideales*	*Neodevrisea*	*Glomeromycotina*
Cystobasidiomycetes	*Metarhizium*	*Oedocephalum*	*Glomus*
Cytospora	*Neopyrenochaeta*	*Peniphora*	*Graphium*
Didymosphaeria	*Occultifur*	*Phaeococcomyces*	*Hyphoderma*
Elsinoe	*Paraophibolus*	*Polysporus*	*Juxtiphoma*
Genolevuria	*Pezicula*	*Pseudorobillarda*	*Massarina*
Hydnocystis	*Pleurotus*	*Simplicillium*	*Meira*
Hyphodontia	*Populocrescentia*	*Stagonospora*	*Moesziomyces*
Kondoa	*Pseudocamarosporium*	*Stephensia*	*Natantipora*
Krasilnikovozyma	*Pulvinula*	*Valsa*	*Neurospora*
Lachancea	*Seimatosporium*	*Vararia*	*Oidiodendron*
Niaceae	*Stachylidium*	*Volutella*	*Oncopodiella*
Paraphaeospharia	*Tetramelas*		*Orbilia*
Protocreopsis	*Wojnowiciella*		*Parasola*
Pseudomicrostroma	*Yarrowia*		*Phytopythium*
Pseudopezicula			*Podospora*
Rachicladosporium			*Preussia*
Rhizophydium			*Pseudodictyosporium*
Scopulariopsis			*Puccinia*
Sigarispora			*Pyrenosphora*
Sphaerostibella			*Pyxidiophora*
Spizellomyces			*Rhizophagus*
Taphrina			*Rhizopus*
Tetrasphaeria			*Septobasidium*
Tiarosporella			*Solicoccozyma*
Torula			*Sordariales_ud*
Triparticalcar			*Sordariomyectes_ud*
Udeniomyces			*Stachybotryna*
Valsalaria			*Terfezia*
Veronaea			*Tremela*
Yunzhangia			*Variocosporellopsis*
			Verticillium
			Zopfiella

*Fungal general identified in a specific sampling moment in both rootstocks

Table S5. SparCC correlation coefficients between taxa in 110 R rootstock.

**Taxon1**	**Taxon2**	**Correlation**
Acremonium	*Cladosporium*	-0.6277
Acremonium	*Curvibasidium*	-0.6304
Apiotrichum	*Basidiomycota_ud*	-0.5922
Apiotrichum	*Curvibasidium*	0.6461
Apiotrichum	*Graphilbum*	0.5808
Apiotrichum	*Heterocephalacria*	0.6986
Aspergillus	*Curvibasidium*	-0.697
Aspergillus	*Peziza*	0.635
Basidiomycota_ud	*Ilyonectria*	0.6903
Basidiomycota_ud	*Peziza*	0.5881
Basidiomycota_ud	*Talaromyces*	0.8084
Cladosporium	*Penicillium*	0.5889
Classicula	*Ilyonectria*	0.6324
Clonostachys	*Curvibasidium*	-0.6
Colletotrichum	*Pleospora*	0.6418
Cryptococcus	*Curvibasidium*	0.678
Curvibasidium	*Diatractium*	0.6466
Curvibasidium	*Heterocephalacria*	0.6458
Curvibasidium	*Lophiostoma*	-0.5832
Curvibasidium	*Phaeomoniella*	-0.5937
Dactylellina	*Tremellomycetes*	-0.5945
Filobasidium	*Rhodotorula*	0.6153
Fitzroyomyces	*Holtermanniella*	0.6373

Graphilbum	*Heterocephalacria*	0.6264
Holtermanniella	*Phaeoacremonium*	-0.5855
Holtermanniella	*Tremellomycetes*	0.6524
Ilyonectria	*Talaromyces*	0.7391
Lophiostoma	*Ochroconis*	0.6123
Naganishia	*Oncopodiella*	0.6293
Naganishia	*Sebacina*	0.6003
Naganishia	*Sordariales_ud*	0.7039
Ochroconis	*Phaeoacremonium*	0.6015
Ochroconis	*Sebacina*	0.664
Oncopodiella	*Rhodotorula*	0.6028
Oncopodiella	*Sebacina*	0.641
Oncopodiella	*Sordariales_ud*	0.8862
Oncopodiella	*Zygosporium*	-0.6271
Penicillium	*Phaeomoniella*	-0.6159
Penicillium	*Zygosporium*	0.7392
Phaeoacremonium	*Sebacina*	0.6378
Phaeoacremonium	*Sordariales_ud*	0.5985
Phaeoacremonium	*Tremellomycetes*	-0.6057
Phaeomoniella	*Sordariales_ud*	0.6232
Psathyrella	*Sordariales_ud*	0.6326
Pseudohyphozyma	*Sordariales_ud*	-0.586
Rhodotorula	*Sordariales_ud*	0.6239
Sebacina	*Sordariales_ud*	0.743
Sebacina	*Zygosporium*	-0.6616
Sordariales_ud	*Zygosporium*	-0.7207

Table S6. SparCC correlation coefficients between taxa in 41 B rootstock.

**Taxon1**	**Taxon2**	**Correlation**
Acremonium	*Clonostachys*	0.778
Acremonium	*Phaeoacremonium*	0.6154
Acremonium	*Rhizoctonia*	0.6869
Acremonium	*Talaromyces*	0.6942
Alternaria	*Neosetophoma*	-0.6607
Apiotrichum	*Basidiomycota_ud*	-0.665
Apiotrichum	*Cladosporium*	0.8515
Apiotrichum	*Curvibasidium*	0.7053
Apiotrichum	*Filobasidium*	0.8224
Apiotrichum	*Holtermanniella*	0.7681
Apiotrichum	*Lophiostoma*	-0.6356
Apiotrichum	*Neofusicoccum*	0.618
Apiotrichum	*Rhizoctonia*	-0.6166
Apiotrichum	*Talaromyces*	-0.658
Apiotrichum	*Tremellomycetes*	0.6849
Aspergillus	*Phaeoacremonium*	0.6264
Aspergillus	*Rhizoctonia*	0.6959
Aspergillus	*Talaromyces*	0.7618
Basidiomycota_ud	*Filobasidium*	-0.6941
Basidiomycota_ud	*Holtermanniella*	-0.659
Basidiomycota_ud	*Rhizoctonia*	0.6304
Basidiomycota_ud	*Talaromyces*	0.7592

Cadophora	*Rhizoctonia*	0.6231
Ceratobasidium	*Pleospora*	-0.6023
Cladosporium	*Filobasidium*	0.7982
Cladosporium	*Fusarium*	-0.6838
Cladosporium	*Holtermanniella*	0.6892
Cladosporium	*Neofusicoccum*	0.604
Cladosporium	*Ramularia*	0.6557
Cladosporium	*Tremellomycetes*	0.7263
Clonostachys	*Dactylonectria*	-0.6386
Clonostachys	*Lophiostoma*	0.6253
Clonostachys	*Phaeoacremonium*	0.6258
Clonostachys	*Rhizoctonia*	0.7043
Clonostachys	*Talaromyces*	0.6498
Cryptococcus	*Filobasidium*	0.6869
Cryptococcus	*Holtermanniella*	0.6743
Cryptococcus	*Rhodotorula*	0.6282
Curvibasidium	*Filobasidium*	0.6462
Dactylonectria	*Tremellomycetes*	0.6179
Diplodia	*Lophiostoma*	0.6095
Diplodia	*Ramularia*	-0.608
Diplodia	*Sordariales_ud*	0.6493
Filobasidium	*Holtermanniella*	0.928
Filobasidium	*Neofusicoccum*	0.6012
Filobasidium	*Rhizoctonia*	-0.606
Filobasidium	*Talaromyces*	-0.7309

Filobasidium	*Tremellomycetes*	0.6139
Fusarium	*Lophiostoma*	0.6038
Fusarium	*Rhizoctonia*	0.6027
Holtermanniella	*Talaromyces*	-0.7167
Lophiostoma	*Peziza*	0.6549
Lophiostoma	*Phaeoacremonium*	0.729
Lophiostoma	*Ramularia*	-0.6426
Lophiostoma	*Rhizoctonia*	0.7863
Lophiostoma	*Sordariales_ud*	0.6635
Lophiostoma	*Talaromyces*	0.6749
Lophiostoma	*Tremellomycetes*	-0.7321
Peziza	*Phaeoacremonium*	0.7316
Peziza	*Ramularia*	-0.6158
Peziza	*Rhizoctonia*	0.6912
Peziza	*Sordariales_ud*	0.8463
Peziza	*Tremellomycetes*	-0.6453
Peziza	*Zygosporium*	-0.6093
Phaeoacremonium	*Rhizoctonia*	0.8381
Phaeoacremonium	*Sordariales_ud*	0.7754
Phaeoacremonium	*Talaromyces*	0.6255
Phaeoacremonium	*Tremellomycetes*	-0.6892
Pleospora	*Sordariales_ud*	-0.6015
Pleospora	*Tremellomycetes*	0.6307
Pleospora	*Zygosporium*	0.6172
Ramularia	*Rhizoctonia*	-0.6391

Ramularia	*Sordariales_ud*	-0.6022
Ramularia	*Tremellomycetes*	0.8212
Rhizoctonia	*Sordariales_ud*	0.6804
Rhizoctonia	*Talaromyces*	0.7892
Rhizoctonia	*Tremellomycetes*	-0.6967
Sordariales_ud	*Tremellomycetes*	-0.7247
Sordariales_ud	*Zygosporium*	-0.721
Tremellomycetes	*Zygosporium*	0.7143

Table S7. Relative proportion (%) of fungal function from sampling moments inferred by FunGuild.

	Pathotroph	Saprotroph	Symbiotroph	Unassigned
110 R				
Sampling moment 1	3.3± 2.0 Bb	44.4±3.2 Aa	48.0±1.6 Aa	4.2±2.5 Ba
Sampling moment 2	2.9±1.8 Cb	55.0±2.0 Aa	35.5±2.1 Bb	6.6±2.0 Ca
Sampling moment 3	10.5±2.9 Cab	51.0±1.5 Aa	35.5±2.3 Bb	3.0±1.7 Ca
Sampling moment 4	14.1±3.5 Ba	43.2±1.8 Aa	36.0±1.7 Ab	7.1±1.4 Ba
41 B				
Sampling moment 1	2.3± 2.5 Bb	46.8±3.0 Aa	47.9±1.7 Aa	3.0±2.3 Ba
Sampling moment 2	1.8±1.3 Cb	57.2±2.2 Aa	37.5±2.2 Bb	3.5±2.3 Ca
Sampling moment 3	11.1±2.7 Cab	52.8±1.8 Aa	33.8±2.0 Bb	2.3±1.8 Ca
Sampling moment 4	15.4±3.3 Ca	48.7±1.9 Aa	32.5±1.4 Bb	3.4±1.4 Ca

Tukey's test at $P < 0.05$ level. Means followed by the same letter do not differ significantly ($P < 0.05$). Capital letters are for comparison of means in the same row. Small letters are for comparison of means in the same column.

Table S8. Compositions and relative abundance (%) of fungal functional groups (guild) inferred by FunGuild

	Symbiotroph			Saprotroph				Pathotroph
110R	Ectomycorrhizal	Endophyte	Lichenized	Wood Saprotroph	Soil Saprotroph	Dung Saprotroph	Undefined Saprotroph	Plant Pathogen
Sampling moment 1	0 b	39.0 ab	3.0 a	43.0 a	2.5 b	0 b	9.0 b	3.5 b
Sampling moment 2	0 b	43.1 a	0 a	42.5 a	0 b	0 b	9.5 b	4.9 b
Sampling moment 3	0 b	38.0 ab	2.0 a	31.8 ab	0.7 b	0 b	17.7 a	9.8 ab
Sampling moment 4	4.8 a	27.4 b	0 a	18.0 b	11.4 a	5.9 a	20.0 a	12.5 a
41B								
Sampling moment 1	0 b	49.8 a	0 a	37.6 ab	1.2 b	0 b	9.1 b	2.3 b
Sampling moment 2	0 b	32.1 ab	0.4 a	47.1 a	0.8 b	0 b	17.2 a	2.4 b
Sampling moment 3	0 b	31.9 ab	0 a	32.7 ab	4.7 a	0 b	20.3 a	10.4 a
Sampling moment 4	4.8 a	26.1 b	0 a	22.2 b	6.7 a	6.1 a	20.0 a	14.1 a

Tukey's test at $P < 0.05$ level. Means followed by the same letter do not differ significantly ($P < 0.05$) among sampling moments within each functional group.

Table S9. Abundance and DNA concentration of *Cadophora luteo-olivacea* in each plant analyzed inferred by NGS and ddPCR, respectively.

Sample ID	Relative abundance (No OTUs)	Absolute abundance (copies/µl)
DG1	891	53
DG2	2,350	27
DG3	1,098	7
DG5	3,149	45
DG6	2,520	35
DG8	443	5.5
DG9	455	13
DG10	65	14.5
DG11	1,262	4
DG12	206	2
DG13	185	1
DG14	401	12
DG15	207	5
DG16	45	13.5
DG17	426	7
DG18	2,411	8
DG19	747	5
DG20	65	9
DG21	13,342	102
DG22	27,709	21
DG23	11,412	167
DG24	12,271	135
DG25	14,692	122
DG26	29,143	44
DG27	15,667	53.5
DG28	65,999	134
DG29	8,765	46
DG30	83,228	177
DG31	9.178	34
DG32	8.594	21
DG33	6.693	16
DG34	6.279	11
DG35	7.310	45
DG36	5.185	22
DG37	5.353	34
DG38	5.800	33
DG39	4.989	17
DG40	11,106	87.5
DG41	26,050	121.5
DG42	67,465	101
DG43	95,891	166
DG44	16,832	110
DG45	18,990	42.5
DG46	65,432	101
DG47	64,790	123.5
DG48	21,709	97
DG49	72,432	141
DG50	21,877	23
DG51	16,665	80
DG52	17,135	75
DG53	17,413	72
DG54	4,729	45
DG55	1,612	55

DG56	4,775	101.5
DG57	863	14
DG58	18,598	45
DG59	3,833	52
DG60	20,339	94
DG61	18,128	44.5
DG62	2,027	17.5
DG63	90	12
DG64	18,478	145
DG65	7,912	33
DG66	545	6.5
DG67	8,626	43
DG68	1,886	53.5
DG69	6,632	52
DG70	884	8.5
DG71	13,409	32.5
DG72	1,805	22.5
DG73	21,574	99
DG74	1,195	6
DG75	7,231	30
DG76	304	12
DG77	347	10
DG78	906	7.5
DG79	5,825	23
DG80	3,906	42

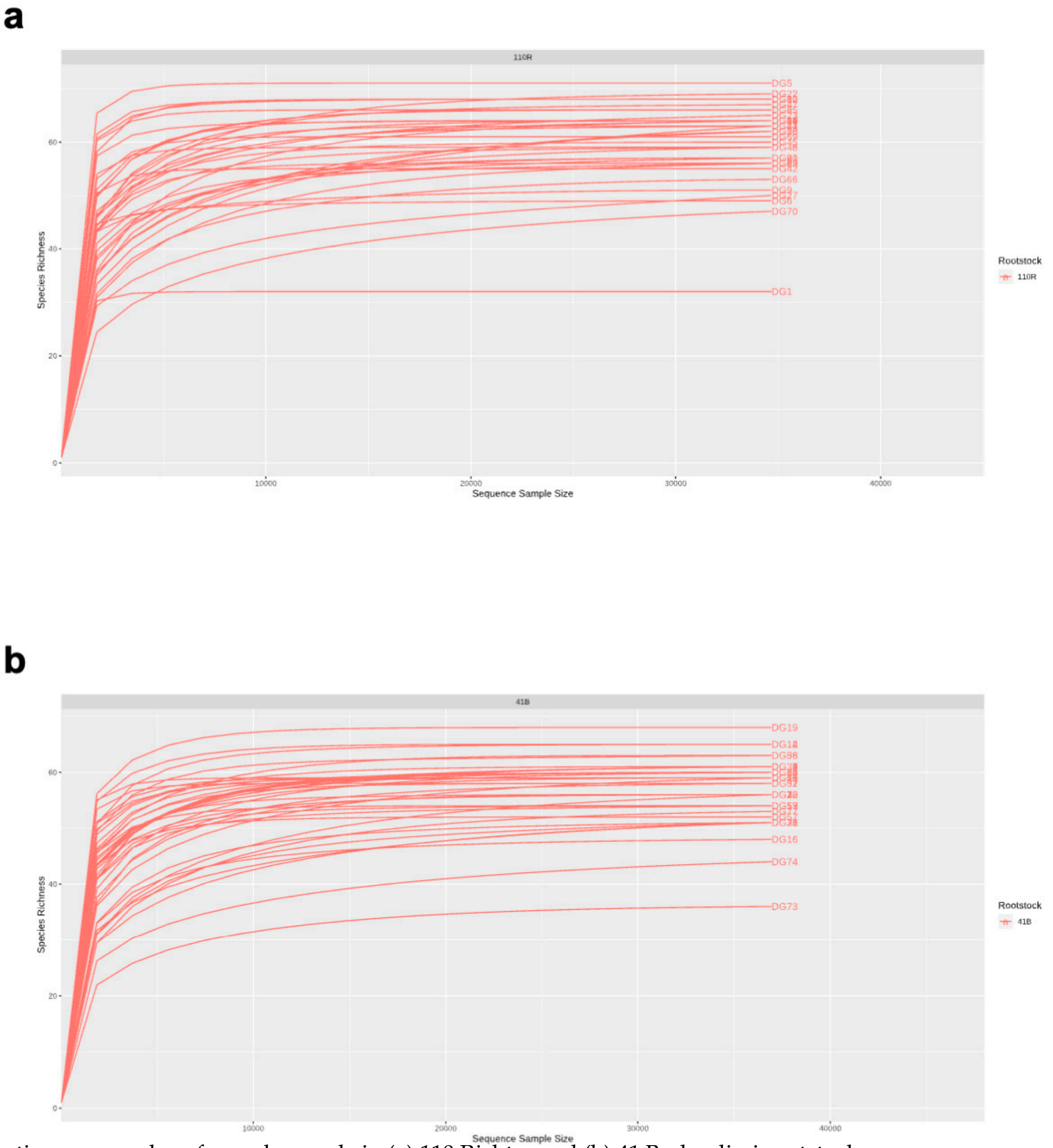

Figure S1. Rarefaction curve values for each sample in (a) 110 Richter and (b) 41 Berlandieri rootstocks.

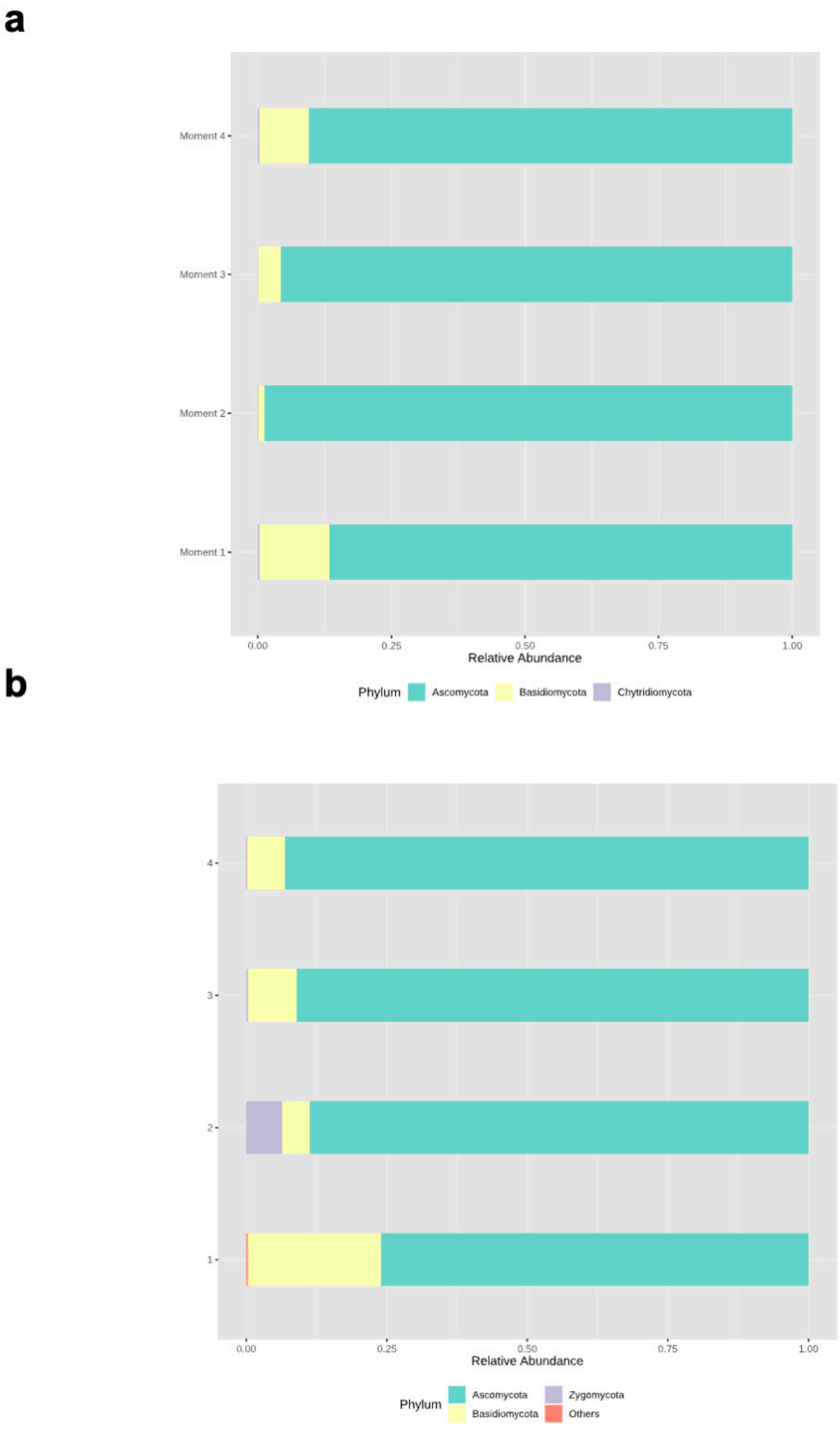

Figure S2. Relative abundance of different fungal phyla in (a) 110 Richter and (b) 41 Berlandieri rootstocks.

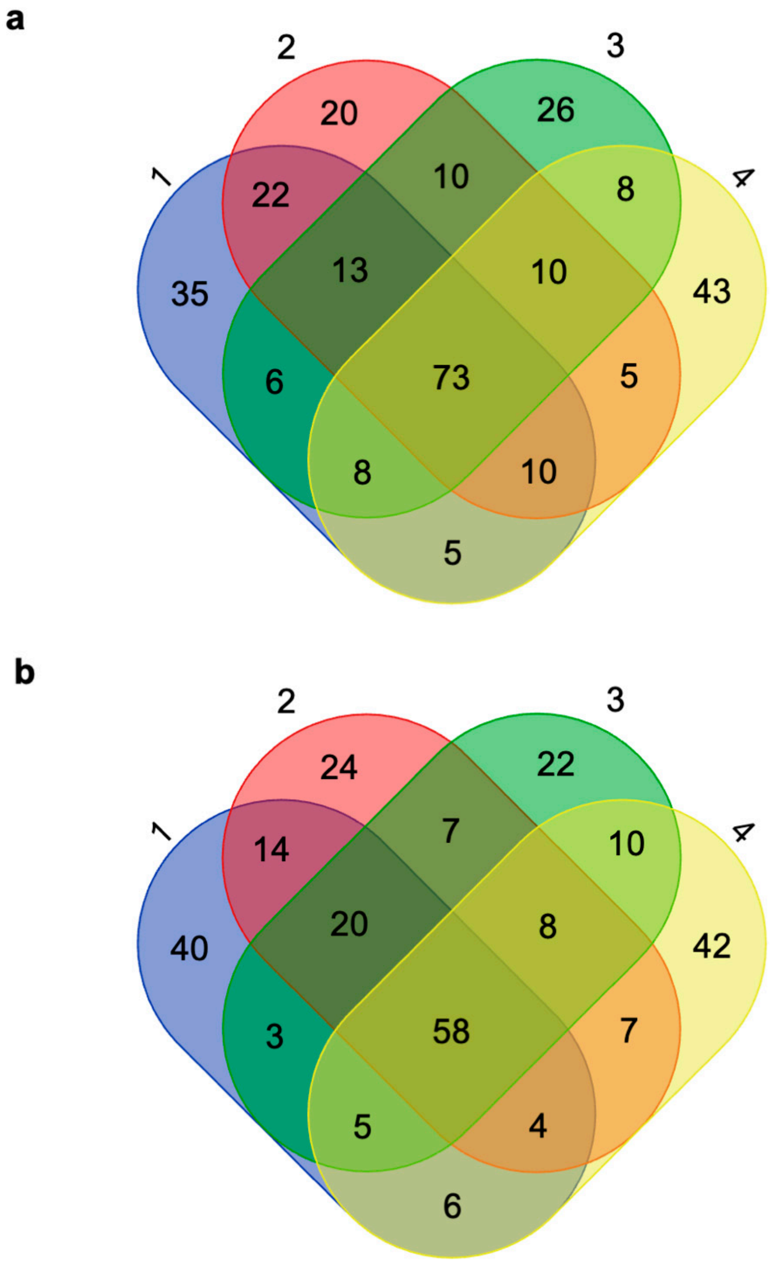

Figure S3. Venn diagram illustrating the overlap of the number of OTUs identified in the fungal microbiota among sampling moments in (a) 110 Richter and (b) 41 Berlandieri rootstocks.

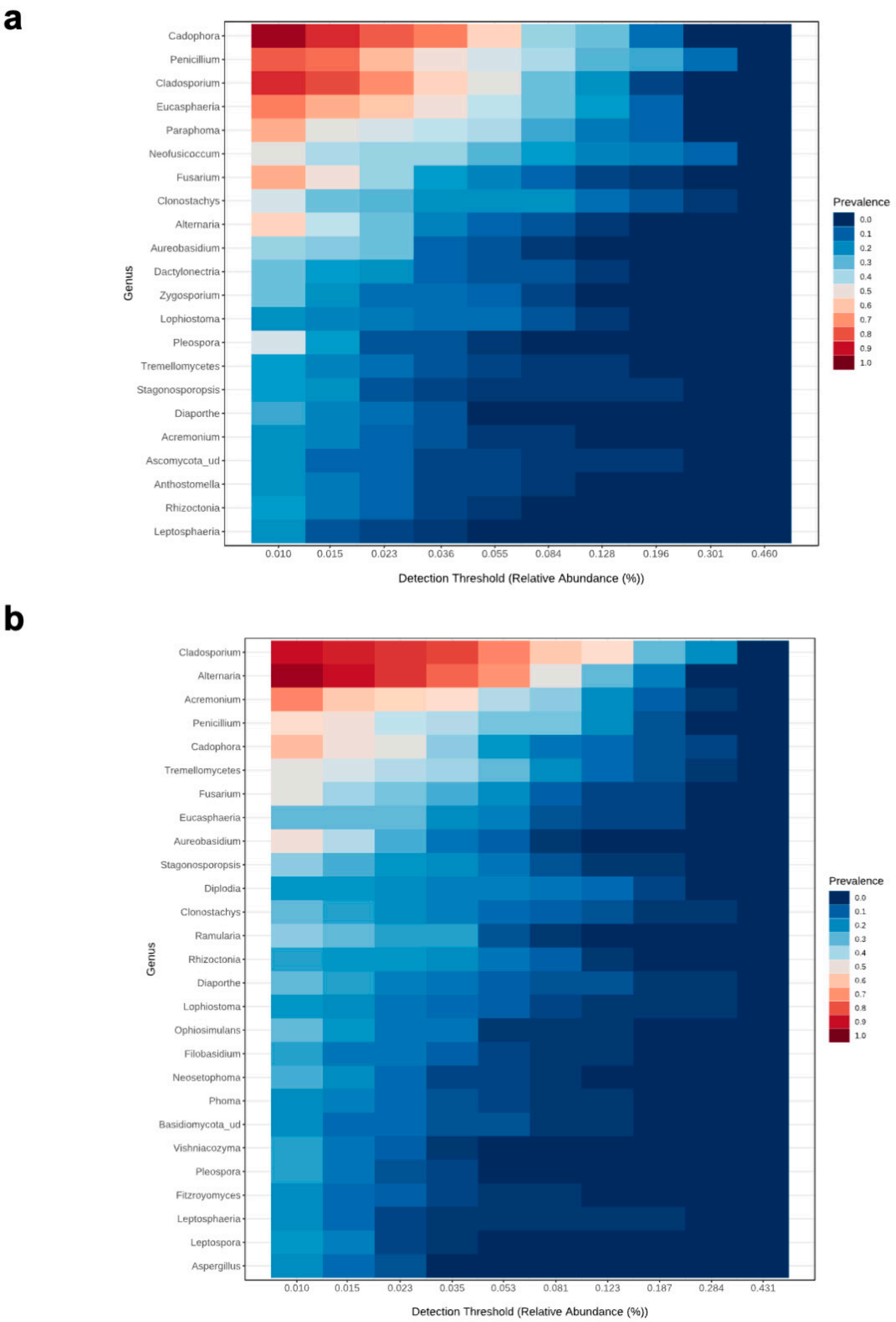

Figure S4. Core microbiome analysis showing a limited number of genera prevalent across all the samples in (a) 110R and (b) 41B rootstocks. Sample prevalence threshold is set up above 20%, and relative abundance threshold is set up above 0.01%. The heatmap colors represent the sample prevalence values.
